# Seroprevalence of anti-SARS-CoV-2 total antibody is higher in younger Austrian blood donors

**DOI:** 10.1007/s15010-021-01639-0

**Published:** 2021-06-16

**Authors:** Lisa Weidner, Verena Nunhofer, Christof Jungbauer, Alexandra Domnica Hoeggerl, Lydia Grüner, Christoph Grabmer, Georg Zimmermann, Eva Rohde, Sandra Laner-Plamberger

**Affiliations:** 1 Austrian Red Cross, Blood Service for Vienna, Lower Austria and Burgenland, Wiedner Hauptstraße 32, 1040 Vienna, Austria; 2 Department for Transfusion Medicine, University Hospital of Salzburg (SALK), Paracelsus Medical University (PMU) Salzburg, Müllner-Hauptstraße 48, 5020 Salzburg, Austria; 3 Team Biostatistics and Big Medical Data, IDA Lab Salzburg, PMU Salzburg, Strubergasse 16, 5020 Salzburg, Austria; 4 Department of Research and Innovation, PMU Salzburg, Strubergasse 16, 5020 Salzburg, Austria; 5 Spinal Cord Injury and Tissue Regeneration Centre Salzburg, PMU Salzburg, Strubergasse 21, 5020 Salzburg, Austria

**Keywords:** Seroprevalence, COVID-19, SARS-CoV-2, Blood donation

## Abstract

**Purpose:**

Frequently the infection with coronavirus 2 (SARS-CoV-2) can be asymptomatic or provoke only mild symptoms. These cases often remain unnoticed, so it is difficult to estimate the actual numbers of infections. Aim of this study was to determine the seroprevalence of anti-SARS-CoV-2 total antibody in Austrian blood donors.

**Methods:**

20,228 blood donors aged between 18 and 72 years resident in four Austrian federal states were screened for anti-SARS-CoV-2 total antibody between 5th of June and 4th of December 2020. To evaluate the impact of sex, age, AB0-blood group and donation period on the anti-SARS-CoV-2 seroprevalence, multiple logistic regression was done.

**Results:**

Our data reveal an anti-SARS-CoV-2 seroprevalence of 2.5% overall, significantly depending on the time point of blood donation: after the first Austrian lockdown the seroprevalence was lower compared to the following months, when the rate was constantly rising. While younger blood donors showed significantly higher seroprevalence, no differences were found concerning sex or AB0 blood group.

**Conclusion:**

Broad testing strategies are required to better determine the number of SARS-CoV-2 infections. Screening blood donors as a representative group for the adult population could be a valid tool to determine the number of recorded and unrecorded cases of SARS-CoV-2 infection.

## Introduction

In December 2019, clusters of patients suffering from pneumonia were reported in China, caused by a novel coronavirus [1]. This enveloped RNA-virus belongs to the family of beta-coronaviruses, causing coronavirus disease 2019 (COVID-19) [2]. This disease resembles severe acute respiratory syndrome (SARS) and Middle East respiratory syndrome (MERS), both caused by two other strains of coronaviruses [3, 4]. Therefore, the World Health Organization (WHO) termed the novel virus “SARS-coronavirus-2 (SARS-CoV-2)” [https://www.who.int/emergencies/diseases/novel-coronavirus-2019/technical-guidance/naming-the-coronavirus-disease-(covid-2019)-and-the-virus-that-causes-it, accessed 03.05.2021]. The majority of SARS-CoV-2 positively tested individuals are asymptomatic or develop mild, flu-like symptoms, such as cough, fever, headache and myalgia. While hyposmia and dysgeusia are described as common symptoms of this virus infection, occasionally dyspnoea, diarrhoea and nausea are reported as well [5]. However, more severe courses of COVID-19 feature acute respiratory distress syndrome (ARDS), sepsis, neurological complications and coagulation disorders among others. These severe courses are often associated with increased age or other comorbidities, such as respiratory diseases, diabetes, cardiovascular diseases or obesity [6]. Currently, the WHO assumes a mortality rate of 3–4% [https://www.who.int/publications/i/item/WHO-2019-nCoV-Sci-Brief-Mortality-2020.1, accessed 03.05.2021].

By May 2021, over 150 million cases of SARS-CoV-2 infections were reported globally, with more than 3 million deaths (COVID-19 Map—Johns Hopkins Coronavirus Resource Center, jhu.edu, accessed 03.05.2021). However, the exact numbers of SARS-CoV-2 infections are difficult to determine. First, varying testing strategies of different countries lead to statistical variations. Second, countries with younger populations report less morbidity and mortality [7]. Furthermore, since the majority of infections cause mild to moderate symptoms or are even asymptomatic, these cases frequently remain unnoticed. Such cases are challenging the prevention of disease dissemination, since the infected individuals might further spread the virus without even noticing. To obtain accurate prevalence rates within the population, which could help governmental institutions and communities to make appropriate decisions to contain the virus dissemination, it might be helpful to determine the prevalence in a subgroup of the population. Blood donors represent a comparatively healthy subgroup of the adult population. The objective of this study was to determine the anti-SARS-CoV-2 total antibody seroprevalence among regular blood donors resident in four different federal states of Austria as a tool to monitor the epidemic within the Austrian population. In sum, 20,228 blood donors were screened between 5th of June and 4th of December 2020.

## Materials and methods

### Sample collection

We studied the serum samples of 20,228 donors, which were collected in the course of voluntary, non-remunerated whole blood donations in four federal states of Austria (Vienna, Lower Austria, Burgenland and Salzburg) from 5th June until 4th December 2020. In these four states live with 4.4 million people about 49.8% of the Austrian population (https://www.statistik.at/web_de/statistiken/menschen_und_gesellschaft/bevoelkerung/index.html, accessed 18.03.2021).

Before donating blood, all donors had a brief health screening and had to complete a written questionnaire, including an informed consent on pathogen screening as a standard part of the blood donation process. Therefore, only individuals that appeared fit to donate at the time of the blood donation and had not reported symptoms of a common cold within the last two weeks or other health issues within a relevant time span before the donation were included. Samples of all blood donors admitted to regular blood donation with signed informed consent on pathogen screening and the use of rest material for research purposes were included. No further preselection (e.g. according to a putative previous SARS-CoV-2 infection) of sample material was done. Concerning demographics of donors, it is important to note that children (< 18 years) and individuals older than 72 years were not included in this study. Every sample represents an individual donor, in sum, 20,228 were included in this study.

### Serological testing

Donor samples were screened for anti-SARS-CoV-2 total antibody (including IgM, IgG and IgA) using Elecsys Anti-SARS-CoV-2 assay (ACOV2) total antibody electrochemiluminescence immunoassay (ECLIA, Roche Diagnostics, Basel Switzerland) using nucleocapsid (N) antigen as target on two independent cobas8000-e801 devices (Roche Diagnostics) according to manufacturer’s instructions. The sensitivity and specificity given by the manufacturer is 100% (≥ 14 days after SARS-CoV-2 NAT testing) and 99.81%, respectively. The results of this semi-quantitative test are based on the sample signal to cut-off ratio, with values < 1.0 corresponding to non-reactive (negative) results and values ≥ 1.0 corresponding to reactive (positive) results. Reactive samples were subjected to a triple repeat measurement and considered positive for anti-SARS-CoV-2 if the repeated measurements were reactive as well. Less than 0.1% of reactive samples showed a non-reactive result in one out of three repeat measurements. To determine the specificity of the ACOV2 test, a subgroup of 82 reactive samples was also tested with Wantai SARS-CoV-2 Ab ELISA (Wantai Biological Pharmacy, Beijing, China) on BEP III analyser (Siemens Healthcare, Erlangen, Germany) according to manufacturer’s instructions. As published earlier, the Wantai SARS-CoV-2 Ab ELISA showed a sensitivity of 98% and a good correlation with virus neutralisation test [8]. Therefore, it was considered as an acceptable confirmation test.

### Data collection and statistical analysis

Data regarding age, sex and AB0-blood group of the donors included were retrieved from the blood bank information systems swisslab (Nexus-swisslab, Berlin, Germany; data from Salzburg) and Edgelab (Edge Laboratories, Lausanne, Switzerland; data from Vienna, Lower Austria and Burgenland). Basic descriptive analyses were conducted using absolute frequencies, percentages, means, and standard deviations, as appropriate. To evaluate the impact of sex, age, AB0 blood group and the blood donation period on the probability of being SARS-CoV-2 positive, multiple logistic regression with FLIC correction for rare events was applied [9, 10]. Besides the above-mentioned variables, the interactions between sex and age as well as sex and AB0-blood group were also included in the model. For assessing statistical significance, the level α < 0.05 was used. All analyses were carried out using the statistical software R version 4.0.2 [11].

## Results

### Seroprevalence rates for anti-SARS-CoV-2 in four Austrian federal states

20,228 regular blood donors were tested for anti-SARS-CoV-2 total antibody between 5th of June and 4th of December 2020 with an overall seroprevalence of 2.5% (= 497 blood donors). It should be noted that within the given period no fixed sample size was aimed on. The four different federal states showed comparable seroprevalence rates with highest numbers in Salzburg (2.7% anti-SARS-CoV-2-positive). However, Salzburg contributed the highest number of blood donors screened, so putatively the seroprevalence of the other states is lower due to smaller numbers of screened donors (Table 1).

**Table 1:** Seroprevalence of anti-SARS-CoV-2 total antibody in Austrian blood donors

		Anti-SARS-CoV-2 positive	Anti-SARS-CoV-2 negative	Total
1	Total: *N* (%)	497 (2.5)	19,731 (97.5)	20,228
2	Sex: *N* (% in subgroup)			
	Women	194 (2.4)	7774 (97.6)	7968
	Men	303 (2.5)	11,957 (97.5)	12,260
3	Blood group: *N* (% in subgroup)			
	0	203 (2.3)	8468 (97.7)	8671
	A	213 (2.7)	7812 (97.3)	8025
	B	53 (2.1)	2455 (97.9)	2508
	AB	28 (2.7)	996 (97.3)	1024
4	Age: mean (standard deviation)	40.2 (14.3)	41.6 (14.4)	41.6 (14.4)
	Age: *N* (% in subgroup)			
	18–25	121 (3.2)	3623 (96.8)	3744
	26–35	87 (2.2)	3896 (97.8)	3983
	36–45	70 (2.0)	3486 (98.0)	3556
	46–55	133 (2.8)	4601 (97.2)	4734
	56–65	81 (2.2)	3535 (97.8)	3616
	> 65	5 (0.8)	590 (99.2)	595
5	Blood donation period: *N* (% in subgroup)			
	05.06.–04.07.2020	31 (1.8)	1647 (98.2)	1678
	05.07.–04.08.2020	80 (1.2)	6702 (98.8)	6782
	05.08.–04.09.2020	68 (2.0)	3307 (98.0)	3375
	05.09.–04.10.2020	62 (2.4)	2507 (97.6)	2569
	05.10.–04.11.2020	76 (3.0)	2437 (97.0)	2513
	05.11.–04.12.2020	180 (5.4)	3131 (94.6)	3311
6	Federal state: *N* (% in subgroup)			
	Burgenland	3 (0.4)	849 (99.6)	852
	Lower Austria	76 (2.3)	3216 (97.7)	3292
	Salzburg	360 (2.7)	13,004 (97.3)	13,364
	Vienna	58 (2.1)	2662 (97.9)	2720

Results are sorted according to sex (2), AB0-blood group (3), age (4), blood donation period (5) and federal state of blood donation (6). Total numbers of blood donors screened are shown in Sect. (Introduction)

In a subgroup of 82 donor sera, which tested positive with Elecsys Anti-SARS-CoV-2, Wantai ELISA reacted positive in 74 cases. As the Wantai ELISA is considered adequate to neutralisation test for its sensitivity, the calculated specificity of the Elecsys Anti-SARS-CoV-2 is 90.24%. The S/CO (ACOV2) for donor sera considered false positive was ranging between 1.1 and 4.11.

### Seroprevalence is not affected by sex and AB0 blood group

**Fig. 1:**
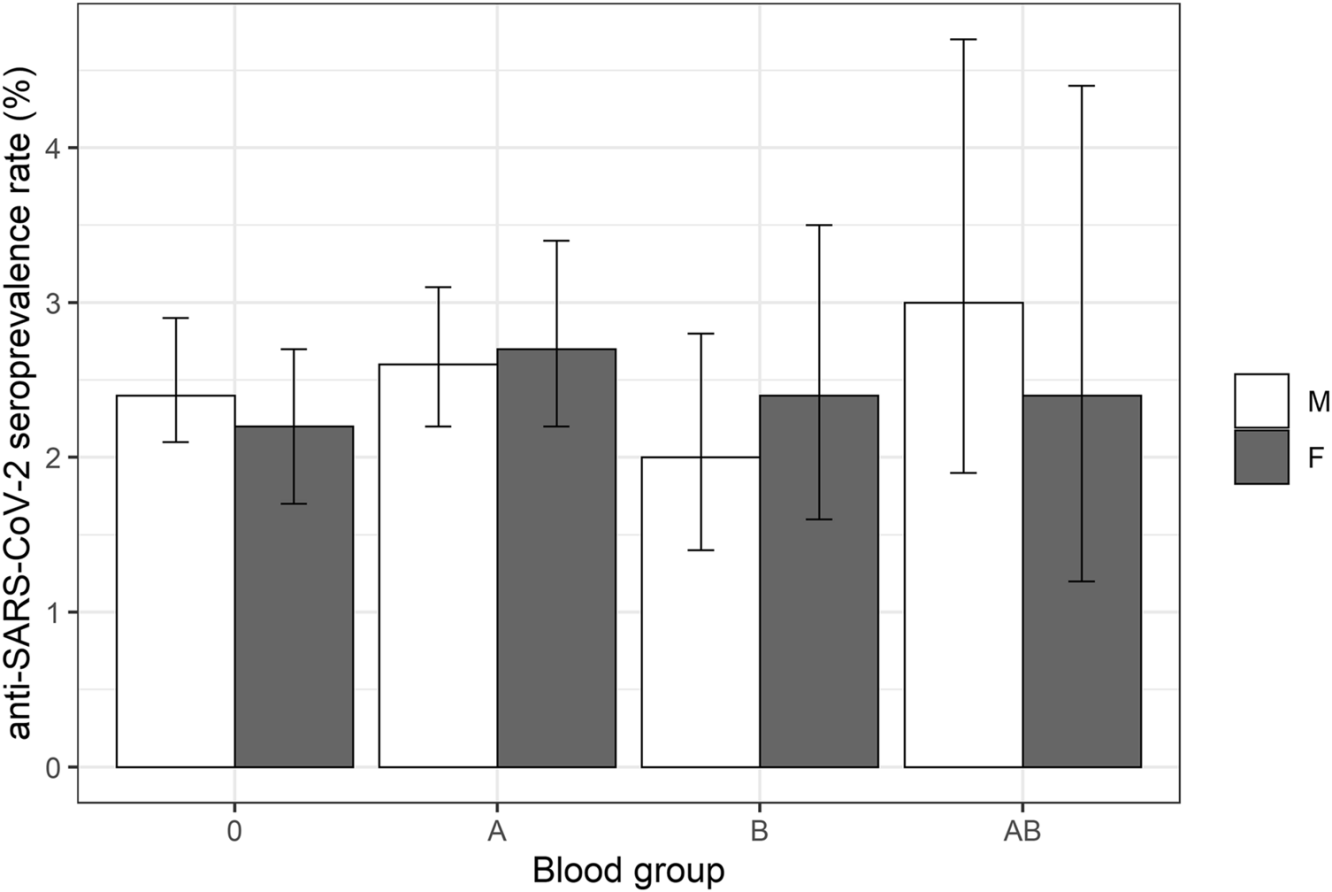
Seroprevalence rates for anti-SARS-CoV-2 total antibody according to AB0-blood group and sex. AB0-blood group and sex do not significantly influence seroprevalence rates. m—male, f—female

No statistically significant differences were observed concerning the anti-SARS-CoV-2 seroprevalence in women (2.4%) or men (2.5%, multiple logistic regression: *p* = 0.9963, see also Tables 1 and 2). Furthermore, we could not find significant differences concerning anti-SARS-CoV-2 seroprevalence between the AB0 blood groups (Tables 1, 2 and Fig. 1).

**Table 2:** Multiple logistic regression analysis with FLIC correction for anti-SARS-CoV-2 seroprevalence rates

Variable	Odds ratio (95% CI)	*p* value
Sex		
Female	1.00 (0.65–1.53)	0.9963
Blood group		
A	1.14 (0.89–1.46)	0.3075
B	0.85 (0.56–1.25)	0.4267
AB	1.36 (0.80–2.18)	0.2443
Age		
18–25	Reference	–
26–35	0.64 (0.44–0.94)	0.0215
36–45	0.78 (0.54–1.13)	0.1888
46–55	0.91 (0.65–1.27)	0.5657
56–65	0.71 (0.49–1.02)	0.0650
> 65	0.22 (0.04–0.63)	0.0026
Blood donation period		
05.06.–04.07.2020	Reference	–
05.07.–04.08.2020	0.62 (0.42–0.96)	0.0321
05.08.–04.09.2020	1.07 (0.70–1.66)	0.7580
05.09.–04.10.2020	1.30 (0.85–2.03)	0.2274
05.10.–04.11.2020	1.62 (1.08–2.51)	0.0199
05.11.–04.12.2020	3.03 (2.09–4.52)	< 0.0001
Sex × age		
Female, 18–25	Reference	–
Female, 26–35	1.07 (0.61–1.88)	0.8058
Female, 36–45	0.42 (0.21–0.79)	0.0074
Female, 46–55	0.85 (0.51–1.42)	0.5391
Female, 56–65	1.00 (0.55–1.79)	0.9969
Female, > 65	2.54 (0.47–15.92)	0.2714
Sex × blood group		
Female, 0	Reference	–
Female, A	1.12 (0.75–1.68)	0.5707
Female, B	1.31 (0.70–2.43)	0.3928
Female, AB	0.88 (0.38–1.98)	0.7652

Data are presented in groups according to sex, blood group, age and donation period. The symbol “ × ” indicates interaction terms

### Age significantly affects the anti-SARS-CoV-2 seroprevalence

**Fig. 2:**
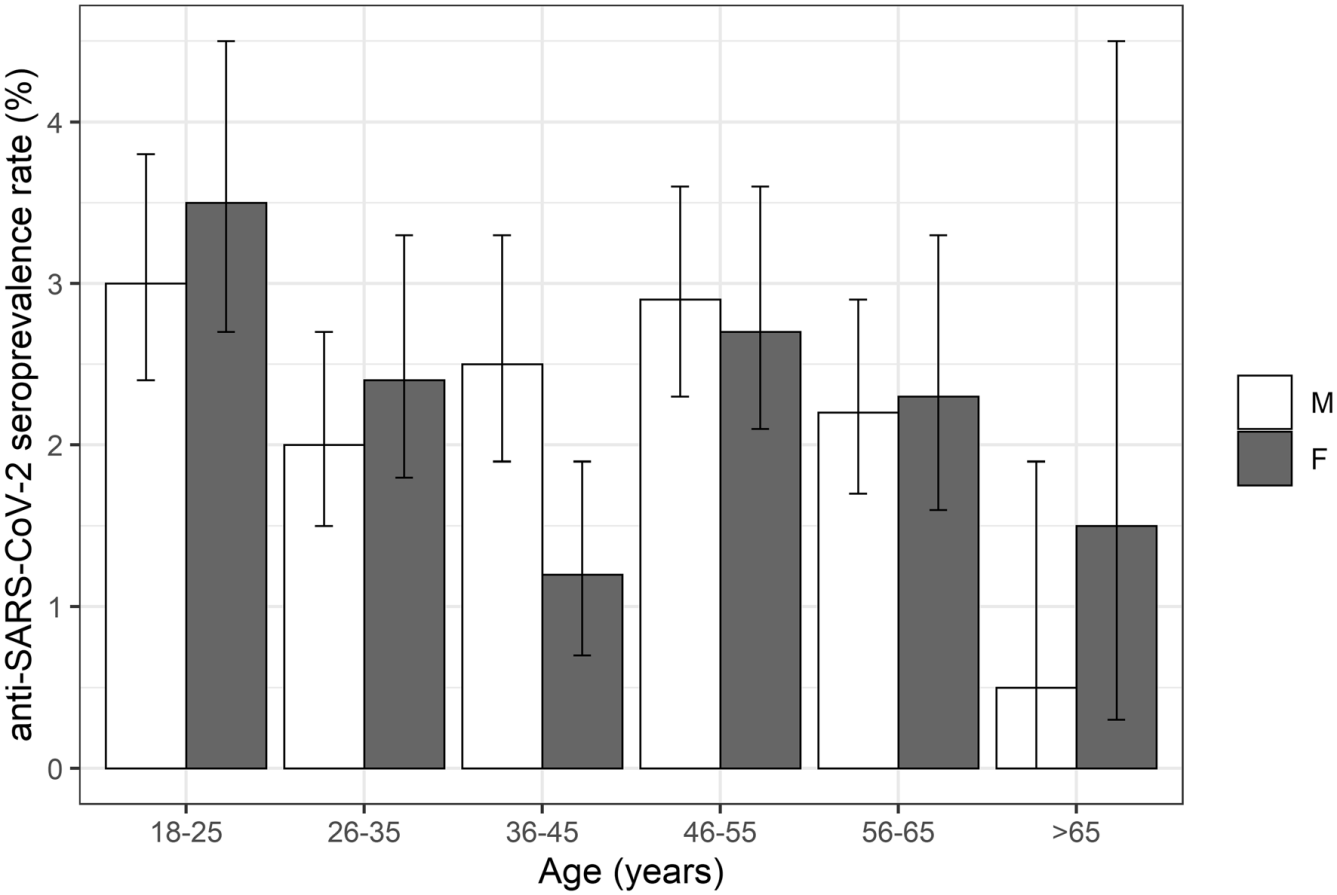
Seroprevalence rates for anti-SARS-CoV-2 total antibody sorted according to sex and age groups. Age groups as indicated, m—male, f—female

Concerning the age of blood donors, we found a significantly higher seroprevalence for donors aged 18–25 compared to other age groups (3.2% compared to 2% as a mean value for the other groups) (Table 1 and Fig. 2). Multiple logistic regression analysis revealed that the age of the blood donors is an important factor: Blood donors show substantially lower odds ratio (OR) when 26–35 years old (0.64, *p* = 0.0215) or older than 56 years (56–65 years: 0.71, *p* = 0.0650; > 65 years: 0.22, *p* = 0.0026) compared to the reference age group 18–25 years, indicating a lower risk for SARS-CoV-2 infections. Blood donors aged 36–55 years show similar odd ratios compared to the reference group 18–25 years (Table 2). In blood donors aged 18–25, seroprevalences were about the same in men and women. Yet, for donors between 36 and 45 years, seroprevalences were markedly lower for women compared to men (Table 2 and Fig. 2). The multiple logistic regression analysis further revealed, that women aged 36–45 show a significantly lower seroprevalence rate compared to women of the reference group 18–25 years (OR 0.42, *p* = 0.0074) (Table 2).

### Blood donation period reflects rising seroprevalence rates after lockdowns

**Fig. 3:**
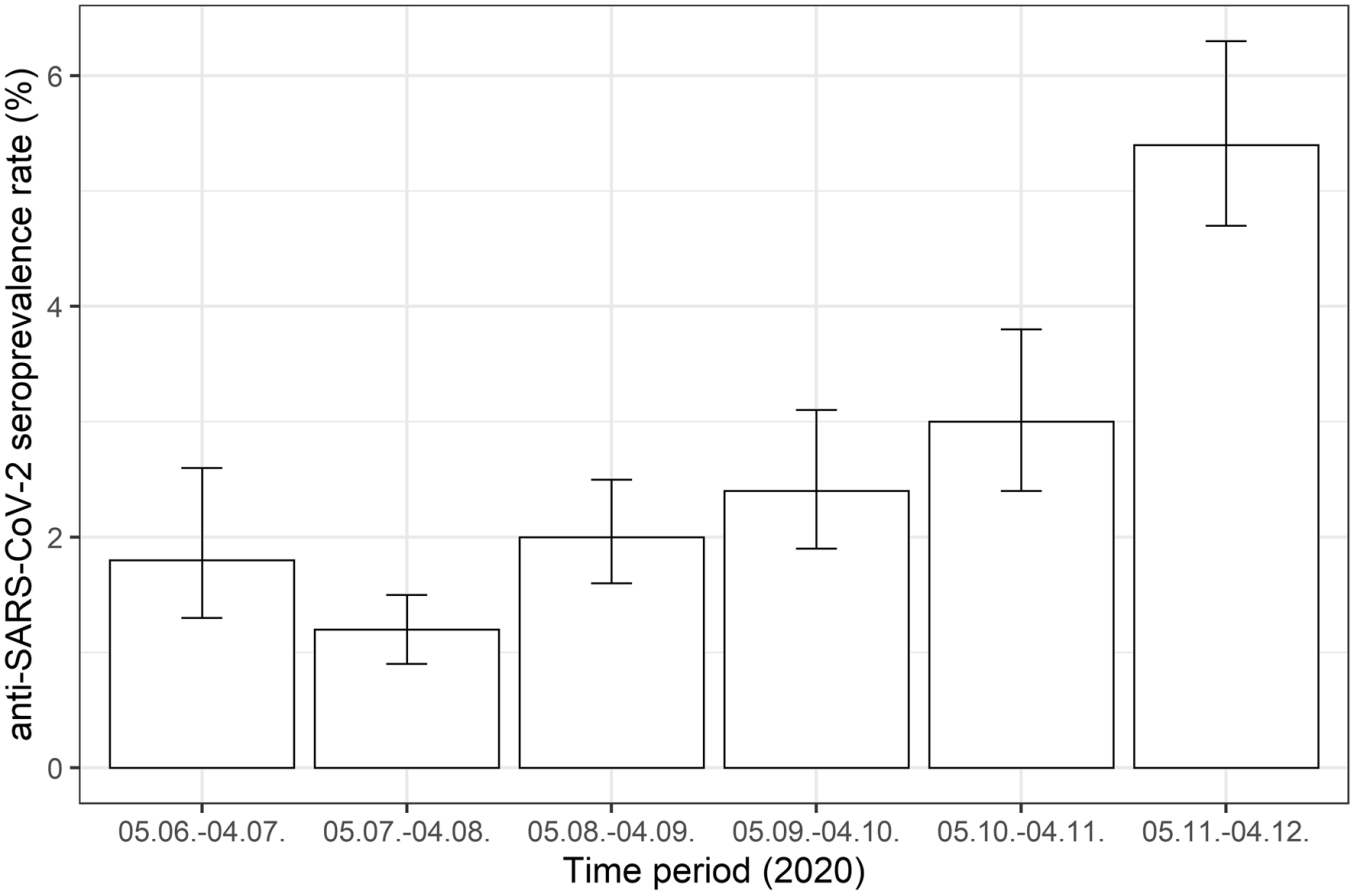
Seroprevalence rates for anti-SARS-CoV-2 total antibody from 05.06. to 04.12.2020

Comparing the different blood donation periods (Tables 1, 2 and Fig. 3), different seroprevalence rates can be observed: At the beginning of this study, the seroprevalence rate was 1.8%. This rate was even reduced in the following period (05.07.–04.08.2020): 1.2% (OR 0.62, CI 95% 0.42–0.96, *p* = 0.0321). After 04.08.2020, seroprevalence rates were constantly rising (2.0% for 05.08.–04.09.2020, 2.4% for 05.09.–04.10.2020, 3.0% for 05.10.–04.11.2020 and 5.4% for 05.11.–04.12.2020). Odd ratios of the multiple logistic regression analysis show rising odds ratios > 1.0, confirming significantly increased seroprevalence rates from 05.10.2020 on (05.10.–04.11.2020: odds ratio 1.62, *p* = 0.0199; 05.11.–04.12.2020: odds ratio 3.03, *p* < 0.0001) (Table 2).

## Discussion

Compared to other European regions, e.g. Lombardy (Italy) with a seroprevalence of 23% [12] or Madrid (Spain) with a seroprevalence rate of 11.5% [13], the overall seroprevalence rate of 2.5% for SARS-CoV-2 determined in our study for the four Austrian federal states is rather low. Our data are comparable to the 2.7% seroprevalence rate determined by a study conducted in the Netherlands [14]. However, in contrast to this study, our data did not reveal significant local differences between the federal states. In the first month of our study, we determined a seroprevalence rate of 1.8%, which even decreased to 1.2% for the period 05.07.–04.08.2020. Our data show an increasing trend of seroprevalence, ascending to 5.4% between 05.11 and 04.12.2020. The low seroprevalence rates at the beginning of our study could be explained by lower infection rates at an earlier stage of the pandemic. A further explanation could be successful preventive interventions, such as the first Austrian lockdown (16.03.–01.05.2020), social distancing and other hygiene measures (e.g. facemasks), as also indicated by others [15, 16]. After the end of the first Austrian lockdown, the SARS-CoV-2 infection rates and thus, as observed in our study, with some delay the seroprevalence rates, were rising again.

We did not find significant differences in the seroprevalence between women and men. A similar observation was described by another study that identified similar prevalence rates between women and men, even though men were more at risk of severe COVID-19 outcomes [17]. Similarly, a review of several population-based studies concluded that the anti-SARS-CoV-2 seroprevalence between men and women is not significantly different [18].

In contrast to other studies [19, 20], which indicated that blood group 0 might be less at risk of being infected, our data did not reveal an impact of AB0-blood groups on anti-SARS-CoV-2 seroprevalence rates. These contradictory results may be explained by different sizes of study groups screened and different test systems and strategies applied. Gallian et al. used neutralizing antibody tests. However, there are studies indicating that not all individuals who recovered from a SARS-CoV-2 infection express detectable levels of neutralizing antibodies [21]. Zietz et al. used swab tests, which were recently reported to differ substantially in analytical sensitivity and specificity [22].

Furthermore, our data revealed significantly higher seroprevalence rates for donors aged 18–25 compared to donors aged 26–35 and donors aged 56 years and older, corroborating the seroprevalence studies from Brazil [23], the Netherlands [14] and Kenya [24]. This could be explained by age-related more active social behaviour during the non-lockdown periods in this youngest group of blood donors. The similar seroprevalence rates for blood donors aged between 36 and 55 years compared to the group aged 18–25 could putatively be related to more social obligations (such as work, caring for children and elderly people) forcing adults aged 36–55 years to have more encounters and therefore putting them at higher risk for viral infections than other age groups. It is important to note that women of the age group 36–45 show a significant lower seroprevalence rate compared to women of the reference group (18–25 years). Furthermore, blood donors aged 65 and older show the lowest seroprevalence rates out of all distinguished age groups. This could be explained by a smaller sample size compared to other age groups, but also by their awareness of their categorisation as a risk group leading to higher precaution in social interactions and concerning hygiene measures.

The presented study has some limitations: It cannot be extrapolated to children and young individuals (< 18 years), adults with more severely impaired health and elderly aged higher than 72 years. In addition, 60% of donors tested positive (resonating with the number of total donors) were male. This might be due to the fact, that men usually have a higher body weight (> 50 kg) and show higher Hb values compared to woman, thus fulfilling the criteria for blood donation more easily. Therefore, the tested population does not fully represent the demographics of the Austrian population with 50.8% females (https://www.statistik.at/web_de/statistiken/menschen_und_gesellschaft/soziales/gender-statistik/demographie/index.html, accessed 25.01.2021). In addition, our testing strategy was to screen for total anti-SARS-CoV-2 antibodies. However, the method used does not allow concluding on the presence of neutralising antibodies against SARS-CoV-2. Neutralisation tests would be necessary to prove the functionality of these antibodies. Furthermore, it should be considered that the seroprevalence in this study group may be overestimated by around 10% based on the calculated specificity of the applied ACOV2 screening test. We therefore also compared our results with an official public data report from the Austrian Social Science Data Archive (AUSSADA) (https://data.aussda.at/dataset.xhtml?persistentId=doi:10.11587/G3C2CS, accessed 30.04.2021), that reports 3.1% seroprevalence on average in mid-November 2020 [95% confidence interval: 2.6–3.5%]. This data set includes people aged 16 and older living in private Austrian households. Even though the seroprevalence of a different group of people was investigated, the seroprevalence of 3.1% matches our finding for blood donors with 3.0% within the same period.

In conclusion, our study reveals that the seroprevalence of anti-SARS-CoV-2 is associated with age, but not with sex or AB0 blood group. Furthermore, the seroprevalence varies according to blood donation period, indicating that lockdowns help to avoid SARS-CoV-2 dissemination. Even though SARS-CoV-2 infections rates are constantly rising worldwide, a seroprevalence rate of 5.4% within a certain population is far too low to reach potential herd immunity within a short period without a complete overload of the health system. Therefore, hygienic measures, social distancing and a vaccination are important and necessary to prevent further SARS-CoV-2 dissemination and thus further burden for health systems and death cases worldwide. Since SARS-CoV-2 infections may show only mild symptoms or be even asymptomatic, many infections might be completely unnoticed. Therefore, screening blood donors as a representative group for the adult population could be a valid tool to determine more exact case numbers of SARS-CoV-2 infections within a population.

## Data Availability

The datasets generated and analysed during the current study are not publicly available due to data protection, but are available in an anonymized manner from the corresponding author on reasonable request.
